# Peri‐ictal imaging abnormalities in non‐convulsive status epilepticus: A systematic review and meta‐analysis comparing magnetic resonance imaging and computed tomography perfusion

**DOI:** 10.1111/epi.18604

**Published:** 2025-08-19

**Authors:** Pilar Bosque‐Varela, Giorgi Kuchukhidze, Wiebke Hahn, Clara Jünemann, Lukas Machegger, Leona Möller, Johannes Pfaff, Eugen Trinka, Susanne Knake, Panagiota‐Eleni Tsalouchidou

**Affiliations:** ^1^ Department of Neurology, Christian Doppler University Hospital, Centre for Cognitive Neuroscience, Member of the European Reference Network EpiCARE Paracelsus Medical University of Salzburg Salzburg Austria; ^2^ Neuroscience Institute, Christian Doppler University Hospital, Centre for Cognitive Neuroscience Paracelsus Medical University of Salzburg Salzburg Austria; ^3^ Epilepsy Center Hessen, Department of Neurology Philipps University Marburg Marburg Germany; ^4^ Department of Neuroradiology, Christian Doppler University Hospital Paracelsus Medical University Salzburg Austria; ^5^ Karl Landsteiner Institute for Clinical Neurosciences Salzburg Austria; ^6^ Second Department of Neurology, Attikon University Hospital National and Kapodistrian University of Athens Athens Greece

**Keywords:** arterial spin labeling, hyperperfusion, perfusion imaging, peri‐ictal MRI abnormalities (PMAs), seizures, status epilepticus

## Abstract

**Objective:**

To assess and compare the detection rates of peri‐ictal abnormalities using magnetic resonance imaging (MRI) and computed tomography perfusion (CTP) in patients with non‐convulsive status epilepticus (NCSE).

**Methods:**

We conducted a systematic literature search in five databases up to February 2025. Studies reporting peri‐ictal MRI abnormalities (PMAs) or cerebral perfusion abnormalities (CPAs) in patients with NCSE were included. Meta‐analyses of proportions were performed using a random‐effects model. Subgroup analyses and meta‐regression were used to compare detection rates across imaging modalities.

**Results:**

Nineteen studies were included (15 MRI, 4 CTP), comprising 562 patients for MRI and 72 for CTP. The pooled detection rate of peri‐ictal abnormalities was 50.0% (95% confidence interval [CI]: 34.0%–65.0%) for MRI and 79.3% (95% CI: 54.3%–92.5%) for CTP. Among the MRI modalities, arterial spin labeling (ASL) demonstrated the highest detection rate at 88.8% (95% CI: 32.9%–99.2%). CTP showed a significantly higher detection rate than MRI (*χ* ^2^ = 3.97, *p* = 0.046); meta‐regression indicated increased odds of detection with CTP (odds ratio [OR] = 4.06, 95% CI: 0.97–16.99, *p* = 0.055). No statistically significant difference was found between ASL and CTP (*χ* ^2^ = 0.22, *p* = 0.636).

**Conclusions:**

CTP demonstrates a higher detection rate than conventional MRI for peri‐ictal abnormalities in patients with NCSE, supporting its utility in rapid diagnosis and differential workup. Among MRI sequences, ASL showed the highest detection rates, highlighting its potential role in the diagnostic assessment of NCSE. Although MRI remains essential for clarifying etiology, its effectiveness in detecting PMA is highly dependent on the sequences used.

**Key points**

1. Computed tomography perfusion showed a higher peri‐ictal abnormality detection rate (79.3%) than magnetic resonance imaging (MRI; 50.0%) in patients with non‐convulsive status epilepticus (NCSE).
2. Among MRI sequences, arterial spin labeling had the highest detection rate for peri‐ictal abnormalities at 88.8%.
3. Perfusion imaging may support NCSE diagnosis, particularly when electroencephalographic findings are inconclusive or symptoms are subtle.

## 1 INTRODUCTION

Status epilepticus (SE) is a medical emergency that can result in long‐term consequences, including neuronal death, if not promptly recognized and treated.^1^ Non‐convulsive SE (NCSE), a subtype of SE, presents a significant diagnostic challenge due to its wide spectrum of clinical manifestations, which may be subtle and easily mistaken for other neurological conditions.^2^, ^3^, ^4^, ^5^, ^6^ Electroencephalography (EEG) remains the gold standard for diagnosing NCSE.^7^, ^8^, ^9^, ^10^, ^11^ However, EEG interpretation in this context is often challenging due to ambiguous patterns, such as the ictal‐interictal continuum (IIC).^11^, ^12^ Despite these diagnostic challenges, early and accurate identification of NCSE is critical, as timely intervention can prevent severe outcomes, including permanent neuronal damage.^13^, ^14^

Neuroimaging has emerged as a potentially valuable tool to support the diagnosis of NCSE, particularly by identifying peri‐ictal magnetic resonance imaging (MRI) abnormalities (PMAs)^15^, ^16^ and hyperperfusion patterns on computed tomography perfusion (CTP).^17^ PMA refers to a range of variable imaging changes in patients with SE, which can be observed across different sequences, such as diffusion‐weighted imaging (DWI), fluid‐attenuated inversion recovery (FLAIR), T2‐weighted imaging (T2WI), contrast‐enhanced imaging, and arterial spin labeling (ASL).^15^, ^18^, ^19^ Modalities such as CTP specifically categorize early dynamic stages of the peri‐ictal period by identifying hyperperfusion patterns.^17^ Although MRI is often the preferred imaging modality due to its superior spatial resolution, providing detailed information on both the etiology and PMAs, CTP offers a more accessible and rapid alternative, especially in emergency settings. However, the detection rates of peri‐ictal abnormalities of these imaging modalities in the context of NCSE remain unexplored, with no comparative data available.

The research question of this systematic review and meta‐analysis was to evaluate and compare the proportion of peri‐ictal abnormalities detected by MRI and CTP in patients with NCSE.

## 2 METHODS

### 2.1 Search strategy and study selection

A systematic literature search was conducted across five electronic databases—PubMed (MEDLINE), Embase, Scopus, Web of Science, and the Cochrane Library—to identify studies evaluating PMAs and cerebral perfusion abnormalities (CPAs) assessed by CTP imaging in adult patients with NCSE. Separate search strategies were employed to independently identify studies reporting PMA on MRI and CPA on CTP in patients with NCSE. Detailed search strategies for each database are provided in the Supplementary Material 1. After retrieving the search results, all records were imported into Mendeley Desktop (version 1.19.8, Elsevier) for de‐duplication. Two independent reviewers (P.B.V. and P.T.) screened the titles and abstracts, followed by a full‐text review of potentially relevant studies. This systematic review and meta‐analysis followed the Preferred Reporting Items for Systematic Reviews and Meta‐Analyses (PRISMA) 2020 guidelines.^20^ The study protocol was registered in the International Prospective Register of Systematic Reviews (PROSPERO; registration number: CRD420251017939).^21^

### 2.2 Eligible studies and participants

Studies were included if they reported the proportion of NCSE patients with PMAs or CPAs. Eligible study designs comprised prospective and retrospective cohort studies, case–control studies, and case series involving more than five adult patients.

Studies were excluded if they did not specifically report PMA or CPA findings for patients with NCSE, particularly when NCSE data could not be distinguished from broader SE populations. Studies focusing solely on convulsive SE, or those describing IIC patterns without a confirmed NCSE diagnosis based on established criteria, were also excluded. In addition, studies were excluded if they involved patients who had not received a diagnosis of NCSE or if patients initially presented with convulsive SE that later progressed to NCSE.

Other exclusion criteria included studies involving pediatric or adolescent populations (defined as patients younger than 18 years of age), case reports, narrative reviews, editorials, letters to the editor, conference abstracts, animal studies, and non–English‐language publications. When multiple studies from the same institution were identified with potentially overlapping data, only the most recent or most comprehensive publication was included to avoid duplication.

### 2.3 Data extraction

Data extraction was conducted using a standardized form to collect key variables, including study characteristics, patient demographics, EEG findings, imaging abnormalities, and clinical outcomes. Two independent reviewers (P.B.V. and P.T.) performed the data extraction in parallel, with any discrepancies resolved through discussion and consensus. Extracted study characteristics included first author, publication year, Digital Object Identifier (DOI), country of origin, institution of the study population, study design, total sample size (N), and the number and proportion of patients diagnosed with NCSE. Demographic data included sex distribution and either mean or median age, depending on availability. The diagnostic criteria used to confirm NCSE—such as the Salzburg^22^, ^23^ and American Clinical Neurophysiology Society (ACNS) criteria^11^–were also documented. In studies where NCSE patients were reported alongside individuals without SE or patients with convulsive SE, data were extracted only for the NCSE subgroup when available. Additional variables, such as the duration of NCSE before MRI acquisition, EEG findings (e.g., lateralized periodic discharges [LPDs]), reversibility of peri‐ictal abnormalities, time to neuroimaging (including whether imaging was performed during ongoing NCSE or afterward), time from neuroimaging to EEG, reported etiology of NCSE, and functional outcomes, including mortality rates, were also extracted when available.

For MRI studies, the number and proportion of PMAs were extracted. Specific MRI sequences were documented individually, including DWI, FLAIR, T2WI, Susceptibility Weighted Imaging (SWI), ASL, T1WI, and contrast‐enhanced perfusion imaging. However, due to limited reporting across studies, subgroup meta‐analyses were feasible for only DWI, FLAIR, and ASL. For studies involving CTP findings, the number and proportion of CPAs in patients with NCSE were also extracted, along with the CTP maps used to identify hyperperfusion.

### 2.4 Quality assessment

The quality of the included studies was assessed using the Newcastle–Ottawa Scale (NOS) for observational studies.^24^ Risk of bias was evaluated across three key domains relevant to the study: selection of study participants, comparability of study groups, and ascertainment of outcomes. Two independent reviewers (L.M. and W.H.) assessed each study and assigned NOS scores based on the predefined criteria. Discrepancies were resolved through discussion or consultation with a third reviewer (C.J.).

### 2.5 Meta‐analysis of imaging abnormalities

Meta‐analyses of proportions were conducted to estimate the pooled prevalence of peri‐ictal abnormalities in patients with NCSE. Analyses included total PMAs identified across all included MRI studies and separate meta‐analyses for specific MRI sequences—DWI, FLAIR, and ASL, when findings were reported individually. A distinct meta‐analysis was also performed for CPA findings reported in CTP imaging studies involving NCSE patients.

All meta‐analyses were conducted using a random‐effects model (DerSimonian–Laird method) to account for inter‐study variability. Study‐specific proportions were logit‐transformed to stabilize variance, and pooled estimates were calculated with 95% confidence intervals (CIs). Heterogeneity was assessed using the *I* ^2^ statistic, *τ* ^2^, and Cochran's *Q* test. Publication bias was evaluated through funnel plots and Egger's test. Forest plots were generated to visualize pooled proportions and corresponding CIs for each analysis.

In addition, sensitivity analyses were performed, including a leave‐one‐out approach to assess the influence of individual studies on the pooled estimates. In this approach, the meta‐analysis was repeated iteratively, excluding one study at a time to determine whether any individual study had a disproportionate effect on the overall findings.

All statistical analyses were performed using R version 4.4.2 (R Core Team, 2024), with the meta and ggplot2 packages.^25^

### 2.6 Comparative analysis between imaging modalities

To compare the rates of peri‐ictal abnormalities detected by MRI and CTP in patients with NCSE, two complementary meta‐analytic approaches were employed: subgroup analysis and meta‐regression.

For the subgroup comparison, the pooled effect sizes (i.e., proportions of abnormal findings) derived from the random‐effects models were compared across imaging modalities. A between‐group heterogeneity test based on Cochran's Q statistic (Q_B_) was used to assess whether the difference in pooled proportions between MRI and CTP was statistically significant.

A meta‐regression analysis was then performed to assess whether imaging modality independently influenced the observed effect sizes across studies. Modality (CTP vs MRI) was included as a binary moderator to evaluate its association with the likelihood of detecting peri‐ictal abnormalities. Study‐level data—specifically the number of abnormal findings and the total number of NCSE patients per study—were used to compute logit‐transformed effect sizes and their corresponding variances. The model estimated the effect of modality on abnormality detection, expressed as an odds ratio (OR; CTP relative to MRI), with 95% CIs.

In addition, a subgroup meta‐analysis was performed to compare CTP and ASL, the two modalities with the highest detection rates. Pooled proportions were calculated using a random‐effects model, and statistical significance of the subgroup differences was tested.

Finally, to explore the impact of MRI timing on detection rates of abnormalities in NCSE, we conducted a subgroup meta‐analysis comparing studies in which MRI was definitively performed within 24 h (“early MRI”) to those performed after 24 h (“late MRI”). Studies were categorized based on clearly reported imaging time points and included only if they reported at least DWI findings.

All statistical analyses were carried out using the metafor package in R version 4.4.2. ORs were calculated by exponentiating the regression coefficients, and 95% CIs were computed to assess statistical significance.

## 3 RESULTS

### 3.1 Search results and study characteristics

Two independent systematic searches were conducted to identify studies reporting peri‐ictal imaging abnormalities in NCSE—one focusing on PMA (MRI) and the other on CPA (CTP). The search covered five databases (MEDLINE, Embase, Scopus, Web of Science, and Cochrane Library), with the final search completed on February 23, 2025. Full search strategies are detailed in Supplementary Material 1.

**FIGURE 1:**
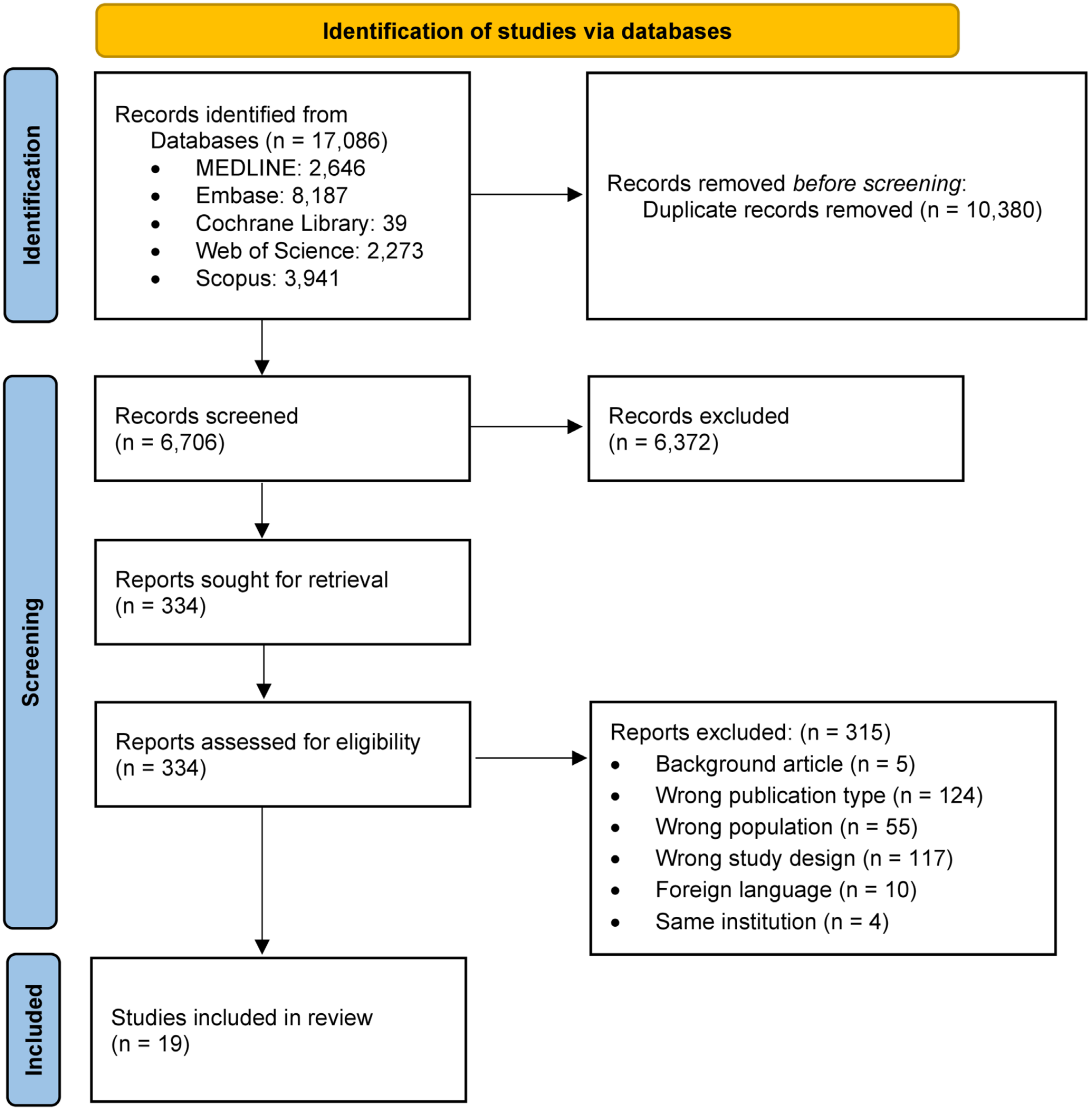
PRISMA flow diagram summarizing the study selection process.

A total of 17 086 records were identified through database searches. Following deduplication and screening, 334 full‐text articles were assessed for eligibility. Ultimately, 19 studies^17^, ^18^, ^19^, ^26^, ^27^, ^28^, ^29^, ^30^, ^31^, ^32^, ^33^, ^34^, ^35^, ^36^, ^37^, ^38^, ^39^, ^40^, ^41^ met all inclusion criteria and were incorporated into the meta‐analysis. A detailed overview of the study selection process is illustrated in the PRISMA flow diagram (Figure 1).^20^ Extracted study characteristics are summarized in Tables 1 and 2, with additional details provided in Supplementary Material 2 (Tables S2.1 and S2.2).

**TABLE 1:** Demographic and clinical characteristics of the MRI population in the included studies.

Author	Year, country	*N*	Total of NCSE	Sex (women, %)	Age^a^	Standardized EEG criteria^b^	PMA				Time from onset to MRI^c^	MRI during ongoing NCSE	Time from EEG to MRI
							Overall	DWI	FLAIR	ASL			
Jaraba Armas et al.	2021, Spain	50	17	76%	Mean = 64 (SD: 16.7)	Yes	10/17 (59%)	8/15 (53%)	NA	2/5 (40%)	NA	NA	NA
Katramados et al.	2010, USA	36	36	47%	Mean = 61.6 (SD: 16)	No	11/36 (31%)	11/36 (31%)	NA	NA	3 days and 12 h	NA	Within 5 days
Sarria‐Estrada et al.	2021, Spain	252	29	NA	NA	Yes	17/29 (59%)	NA	NA	NA	NA/ 123 h (76.8–183.8) h^d^	14/33 (42%)^d^	123 h (IQR 76.8–183.8)^d^
Chen et al.	2023, China	64	27	48%	NA	Yes	12/27 (44%)	4/12 (33%)	4/12 (33%)	NA	NA	NA	NA
Ohtomo et al.	2020, Japan	28	21	62%	Mean = 77.7 (SD: 26)	Yes	21/21 (100%)	NA	NA	21/21 (100%)	Max 24 h (1–24 h)	21/21 (100%)	Max 23 h (1–23 h)
Giovannini et al.	2018, Austria	277	168	NA	NA	Yes	18/168 (11%)	14/16 (44%)	11/16 (34%)	NA	NA/ within 30 days^d^	97/277 (35%)^d^	NA
Shimogawa et al.	2017, Japan	15	15	60%	Median = 69 (range: 32–93)	No	15/15 (100%)	11/15 (73%)	NA	15/15 (100%)	1 hour‐ 2 months	NA	NA
Requena et al.	2019, Spain	60	38	NA	NA	Yes	NA	22/38 (58%)	15/38 (39%)	NA	NA/ 86.5 (42.3–145.3) h^d^	23/60 (38%)^d^	NA
Guven et al.	2025, Turkey	44	13	NA	NA	Yes	12/13 (92%)	12/13 (92%)	0/13 (0%)	NA	NA	NA	NA
Jabeen et al.	2017, India	30	15	27%	Mean = 53.73 (SD: 17.5)	No	10/15 (67%)	10/15 (67%)	NA	NA	24 h	NA	NA
Azman et al.	2020, Turkey	28	16	44%	Mean = 64.7 (SD: 13.4)	Yes	4/16 (25%)	3/16 (19%)	NA	NA	NA	NA	NA
Verma et al.	2016, Switzerland	16	14	64%	Mean = 64.5 (SD: 17.6)	No	8/14 (57%)	8/14 (57%)	NA	NA	NA/<1 h^d^	16/16 (100%)^d^	NA
Bonduelle et al.	2022, France	307	65	NA	NA	Yes	16/65 (25%)	NA	NA	NA	24 h (0–4.8)	NA	NA
Hormigo et al.	2004, USA	8	8	50%	Median = 62 (range: 39–80)	No	4/8 (50%)	3/8 (37%)	3/8 (37%)	NA	NA	NA	NA
Cornwall et al.	2022, Denmark	101	80	NA	NA	Yes	19/80 (24%)	NA	NA	NA	NA/ 24 h‐5 days^d^	NA	NA

Abbreviations: ASL, arterial spin labeling; DWI, diffusion‐weighted imaging; EEG, electroencephalography; FLAIR, fluid‐attenuated inversion recovery; MRI, magnetic resonance imaging; NA, not available or not reported; NCSE, non‐convulsive status epilepticus; PMA, peri‐ictal MRI abnormality.

^a^ Age is expressed either as median (interquartile range, or range) or mean (standard deviation), as reported by the original study.

^b^ Criteria for NCSE diagnosis based on Salzburg or American Clinical Neurophysiology Society (ACNS) definitions, if specified.

^c^ Time to MRI is expressed in hours (h), months or days either as median or mean, as per the original data.

^d^ Whole cohort.

**TABLE 2:** Demographic and clinical characteristics of the CTP population in included studies.

Author	Year, country	N	Total of NCSE	Sex (% women)	Age^a^	Standardized EEG criteria^b^	Hyperperfusion	Time from onset to CTP^c^	CTP during ongoing NCSE	Time from CTP to EEG	CTP maps
Merli et al.	2024, Italy	77	21	43%	Mean = 79 (SD:11.1)	Yes	20 (95.2%)	559 min (±308)	21/21 (100%)	94 (± 172.9)	MTT, CBF, CBV, Tmax, rTmax
Gonzalez‐Martinez et al.	2021, Spain	284	21	NA	NA	NA	11 (52%)	176 min (±156)^d^	NA	NA	CBF, CBV, Tmax
Giovannini et al.	2021, Italy	21	21	48%	Median = 72 (range: 56–90)	Yes	18 (85.7%)	Median: 3 h (range: up to 7 h; known in 16/21, unknown in 5 due to wake‐up symptoms)	21/21 (100%)	≤3 h in 71% (15/21 patients); 4–12 h in 6/21 patients	CBF, CBV, MTT
Hauf et al.	2009, Switzerland.	19	9	78%	Mean = 67.89 (SD: 9.2)	No	7 (77.8%)	NA	9/9 (100%)	<2 h	CBF, CBV, MTT

Abbreviations: CBF, cerebral blood flow; CBV, cerebral blood volume; CTP, computed tomography perfusion; EEG, electroencephalography; MTT, mean transit time; NA, not available or not reported.; NCSE, non‐convulsive status epilepticus; rTmax, reverse time to maximum; Tmax, to maximum.

^a^ Age is expressed either as median (interquartile range, or range) or mean (standard deviation).

^b^ Criteria for NCSE diagnosis based on Salzburg or ACNS definitions, where specified.

^c^ Time to CTP is similarly expressed in min either as median or mean, as per the original data.

^d^ Whole cohort.

### 3.2 Quality assessment/risk of bias

The methodological quality of the included studies was assessed using the NOS for observational studies. Risk of bias was evaluated across three domains: selection of study participants, comparability of study groups, and ascertainment of imaging and clinical outcomes. Overall, most studies exhibited moderate to high methodological quality, with the majority performing well in participant selection and outcome assessment. However, comparability across studies was often limited due to variations in diagnostic criteria for NCSE and differences in imaging protocols. A detailed summary of individual NOS scores and domain‐specific evaluations is provided in Supplementary Material 3.

### 3.3 Findings from the meta‐analysis of MRI studies

**FIGURE 2:**
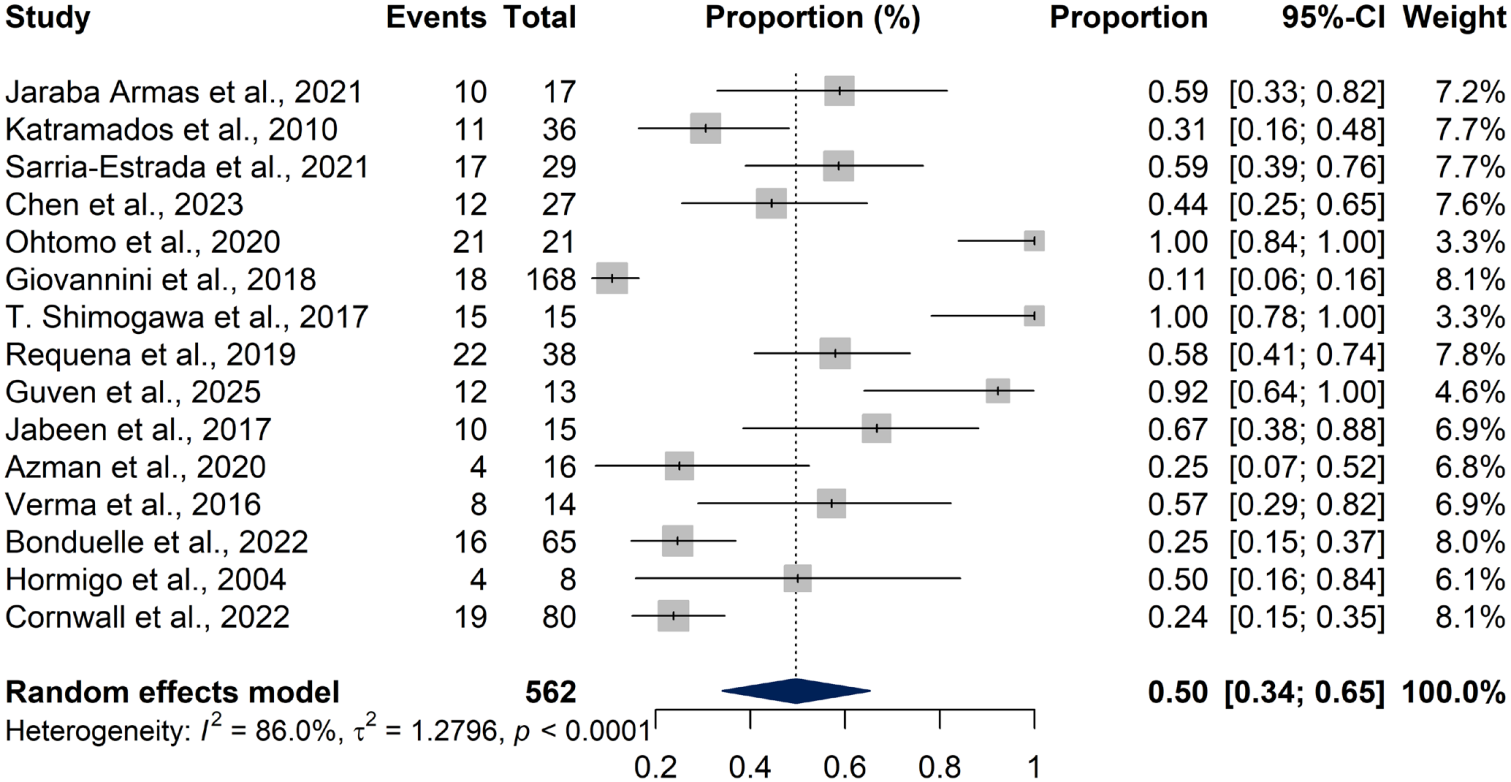
Forest plot showing the pooled proportion of peri‐ictal magnetic resonance imaging (MRI) abnormalities (PMAs) across 15 studies involving patients with non‐convulsive status epilepticus (NCSE). Each horizontal line represents the 95% confidence interval (CI) for the proportion of PMAs in an individual study, and the diamond at the bottom represents the pooled estimate calculated using a random‐effects model.

A total of 15 studies were included in the meta‐analysis of MRI findings, encompassing 562 patients diagnosed with NCSE. The pooled proportion of PMAs was 50.0% (95% CI: 34.0%–65.0%), based on a random‐effects model (*I* ^2^ = 86%, *τ* ^2^ = 1.2796, *Q* = 100.79, *p* < 0.0001), indicating substantial heterogeneity among studies (Figure 2).

**FIGURE 3:**
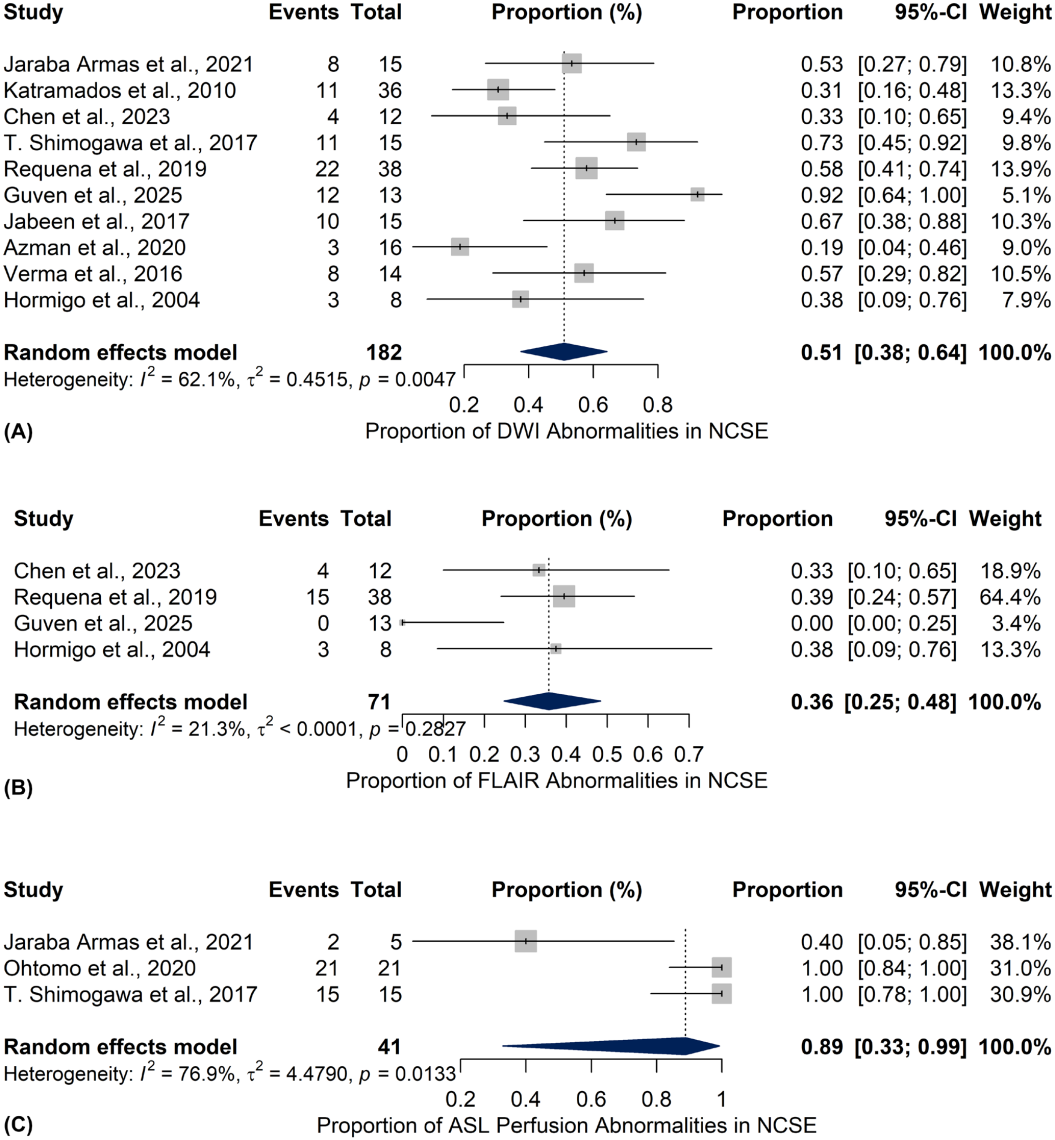
Forest plots showing the pooled proportion of abnormal MRI findings across different imaging sequences in patients with non‐convulsive status epilepticus (NCSE): Diffusion‐weighted imaging (DWI) findings across 10 studies (3A), fluid‐attenuated inversion recovery (FLAIR) findings across 4 studies (3B), and arterial spin labeling (ASL) findings across 3 studies (3C). Each horizontal line represents the 95% confidence interval (CI) for the proportion of abnormalities detected in an individual study, and the diamond at the bottom of each plot indicates the pooled estimate calculated using a random‐effects model.

To investigate modality‐specific variability, separate proportion meta‐analyses were conducted for individual MRI sequences. DWI, assessed across 10 studies, yielded a pooled abnormality proportion of 50.95% (95% CI: 37.56%–64.21%) with moderate heterogeneity (*I* ^2^ = 62.1%, *τ* ^2^ = 0.4515, *Q* = 23.74, *p* = 0.0047) (Figure 3A). For FLAIR, data from four studies showed a pooled abnormality rate of 35.74% (95% CI: 24.81%–48.38%), with low heterogeneity (*I* ^2^ = 21.3%, *Q* = 3.81, *p* = 0.2827) (Figure 3B). In contrast, ASL findings, drawn from three studies, revealed a considerably higher pooled abnormality proportion of 88.83% (95% CI: 32.86%–99.23%), although with substantial heterogeneity (*I* ^2^ = 76.9%, *τ* ^2^ = 4.4790, *Q* = 8.64, *p* = 0.0133) (Figure 3C).

#### 3.3.1 Heterogeneity and publication bias in MRI studies

Substantial heterogeneity was observed (*I* ^2^ = 86%), indicating notable variability across the included studies. Publication bias was assessed using a funnel plot, which demonstrated some asymmetry, suggesting potential small‐study effects or heterogeneity‐driven variation. However, Egger's test (*t *= 1.10, *df* = 13, *p* = 0.2926) did not indicate statistically significant asymmetry, suggesting no strong evidence of publication bias (Supplementary Material 4).

#### 3.3.2 Sensitivity analysis of MRI studies

A leave‐one‐out sensitivity analysis was performed to determine whether any single study disproportionately influenced the pooled estimate. The results showed that the pooled proportion remained consistent across iterations, with only minor variations. The largest shift occurred when Guven et al. (−0.172) and Ohtomo (−0.178) were excluded, slightly reducing the pooled estimate. Conversely, excluding Cornwall et al. (0.093) or Giovannini (0.091) slightly increased the estimate. However, all CIs overlapped, indicating that no single study significantly altered the overall findings. This reinforces the robustness of the meta‐analysis, despite the observed heterogeneity (*I* ^2^ = 86%). The detailed analysis is presented in Supplementary Material 5.

### 3.4 Findings from the meta‐analysis of CT studies

**FIGURE 4:**
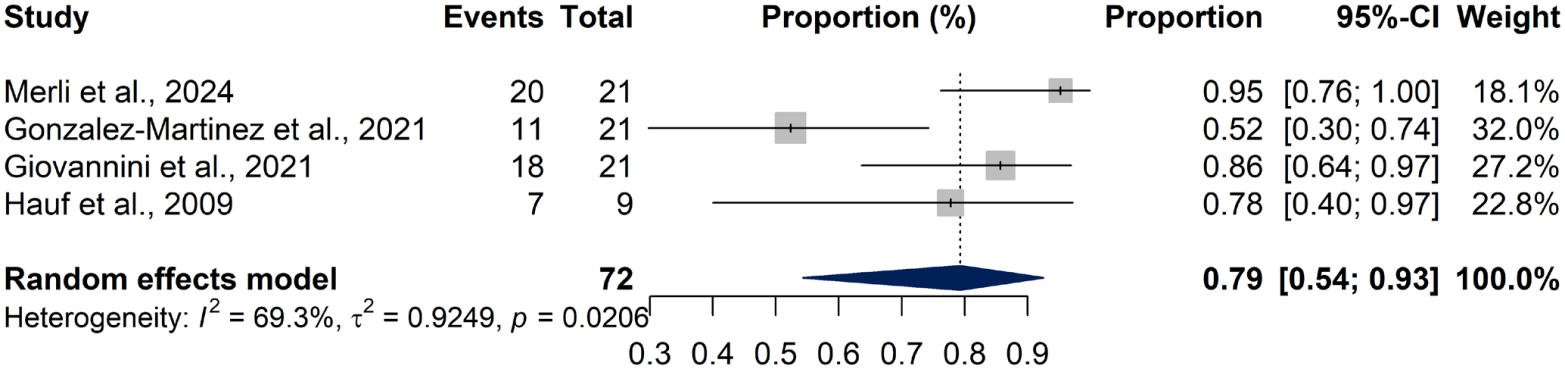
Forest plot showing the pooled proportion of hyperperfusion findings detected via computed tomography perfusion (CTP) imaging in patients with non‐convulsive status epilepticus (NCSE). Each horizontal line represents the 95% confidence interval (CI) for the proportion reported by each study, and the diamond represents the pooled estimate from a random‐effects model.

The meta‐analysis included four studies reporting CTP findings in a total of 72 NCSE patients. The pooled proportion of patients exhibiting hyperperfusion was 79.3% (95% CI: 54.3%–92.5%), based on a random‐effects model (Figure 4). The proportion of hyperperfusion varied across studies, ranging from 52.4% to 95.2%. Heterogeneity was moderate to high (*I* ^2^ = 69.3%), indicating study variability (*τ* ^2^ = 0.9249, *p* = 0.0206).

### 3.5 Comparison between imaging modalities

**FIGURE 5:**
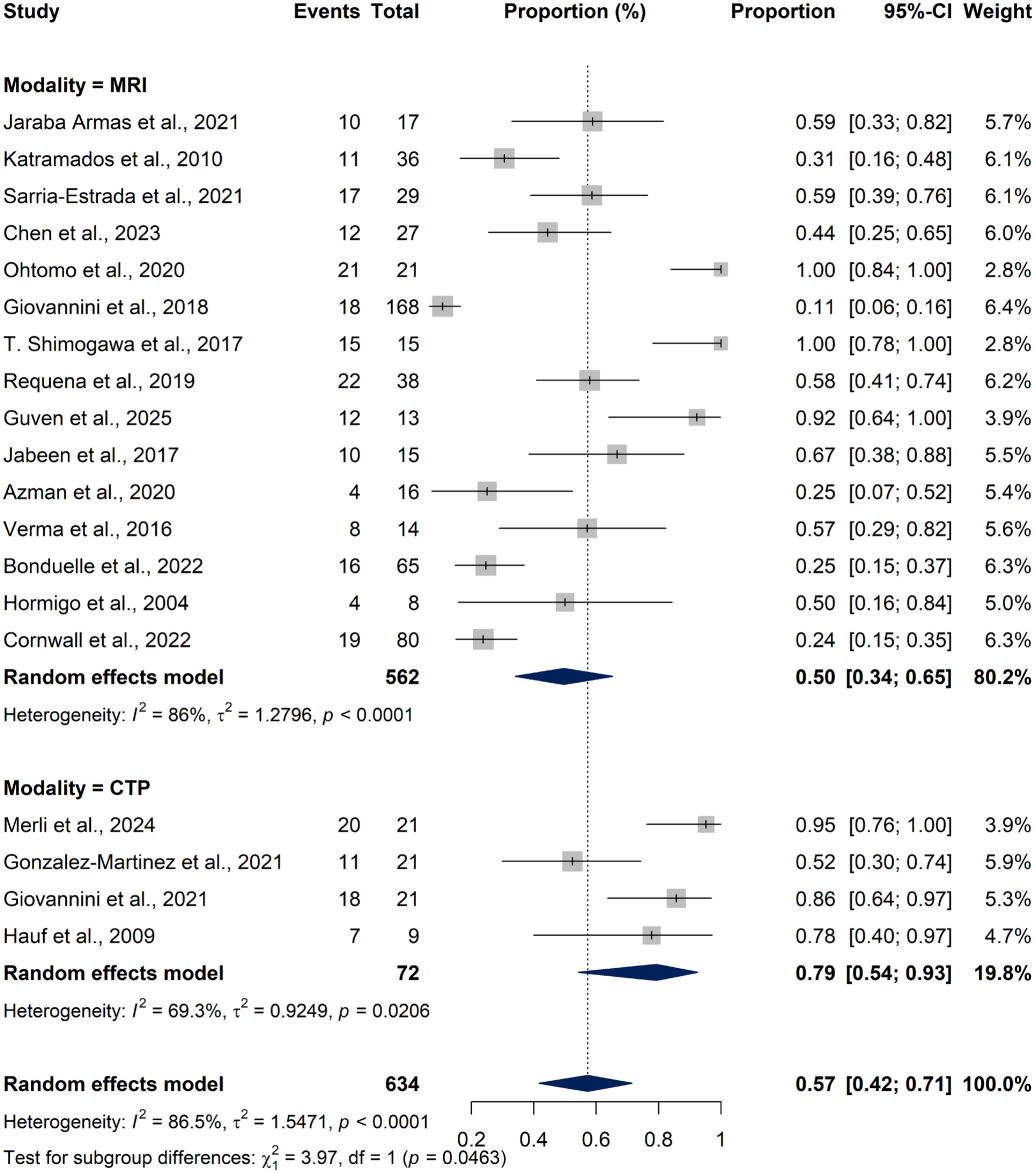
Forest plot presenting subgroup meta‐analysis comparing the pooled proportion of imaging abnormalities detected by magnetic resonance imaging (MRI) vs computed tomography perfusion (CTP) in patients with non‐convulsive status epilepticus (NCSE). The overall pooled estimate (bottom diamond) is provided for visual completeness. Horizontal lines indicate 95% confidence intervals (CIs), and squares represent study‐specific proportions. The test for subgroup differences was statistically significant (*χ* ^2^ = 3.97, *p* = 0.0463), indicating a higher sensitivity of CTP compared to MRI in identifying peri‐ictal abnormalities.

To compare the detection rates of peri‐ictal abnormalities in NCSE between MRI and CTP, both subgroup meta‐analysis and meta‐regression were conducted (Figure 5). The subgroup analysis revealed a significantly higher pooled proportion of imaging abnormalities in CTP studies (0.79, 95% CI: 0.54–0.93) compared to MRI studies (0.50, 95% CI: 0.34–0.65), with a statistically significant difference between the two groups (*χ* ^2^ = 3.97, *p* = 0.046). Complementary meta‐regression modeling, using modality as a moderator, supported this finding by showing a trend toward increased odds of abnormal findings in CTP (OR = 4.06, 95% CI: 0.97–16.99, *p* = 0.055). Although the latter result narrowly missed statistical significance, the consistency between analyses suggests that CTP was associated with a higher detection rate of peri‐ictal abnormalities compared to MRI.

**FIGURE 6:**
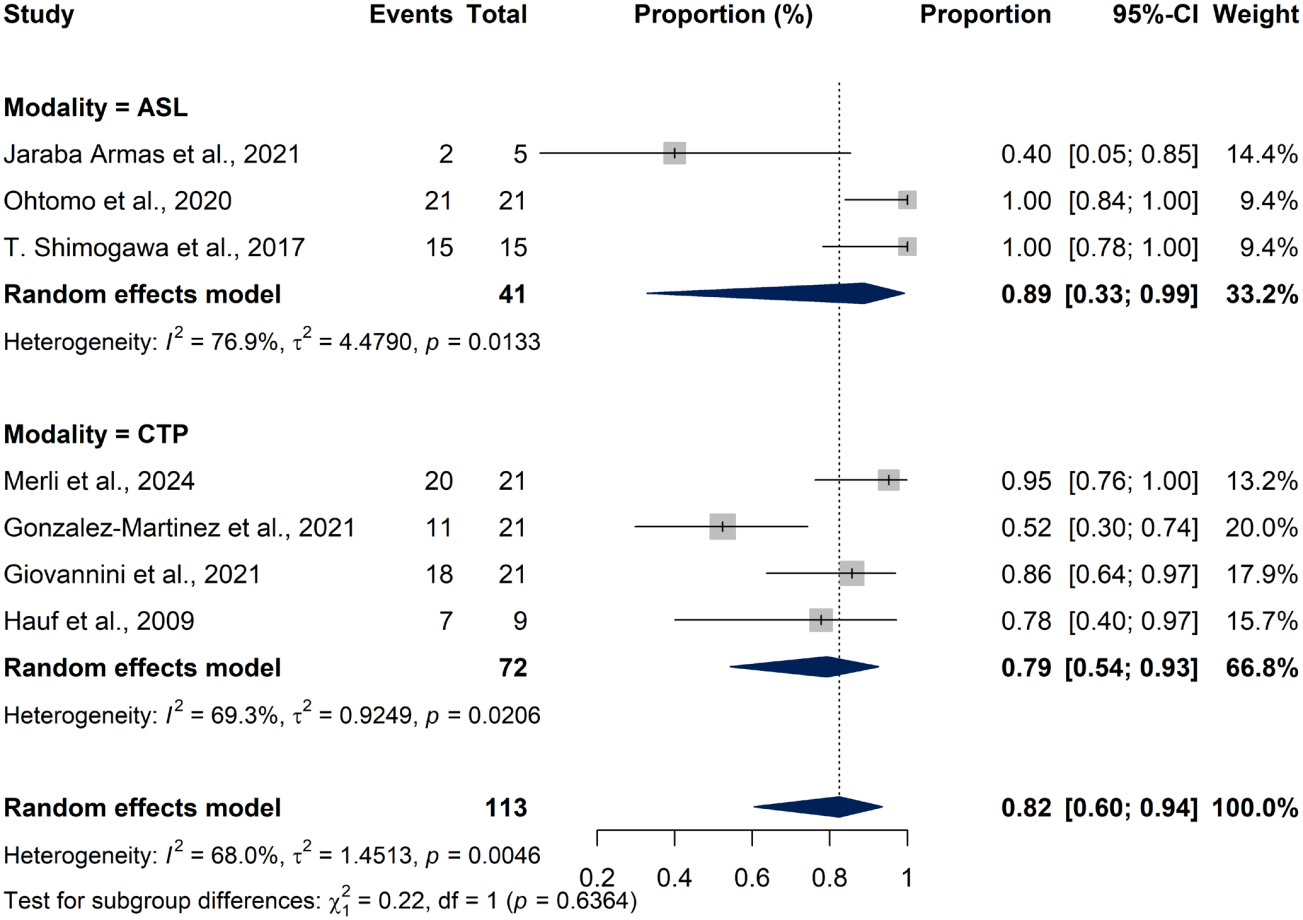
Forest plot comparing the proportion of abnormal peri‐ictal imaging findings between arterial spin labeling (ASL) and computed tomography perfusion (CTP) modalities in patients with NCSE. Each square represents an individual study estimate, with square size reflecting study weight, and horizontal lines indicating 95% confidence intervals. Blue diamonds represent pooled random‐effects estimates for each subgroup and the overall model.

A secondary subgroup comparison between CTP and ASL showed no significant difference (*χ* ^2^ = 0.22, *p* = 0.636), suggesting comparable detection rates (Figure 6). Although ASL exhibited a higher pooled estimate for identifying peri‐ictal abnormalities, the difference between ASL and CTP did not reach statistical significance (*χ* ^2^ = 0.22, *p* = 0.636), suggesting that both perfusion‐based modalities demonstrated similarly high detection rates for peri‐ictal abnormalities in NCSE.

Finally, the subgroup analysis comparing MRI timing included studies classified as “early MRI” (*n* = 3; Bonduelle et al., Verma et al., Jabeen et al.), in which imaging was performed within 24 h of status onset, and “late MRI” (*n* = 3; Katramados et al., Sarria‐Estrada et al., Requena et al.), in which imaging was performed after 24 h. The pooled detection rate of peri‐ictal abnormalities on MRI was 46.9% (95% CI: 21.9%–73.4%) in the early group and 48.8% (95% CI: 31.0%–66.9%) in the late group. Heterogeneity was substantial in both subgroups (*I* ^2^ = 82.6% and 71.1%, respectively), and the test for subgroup differences was not statistically significant (*Q* = 0.01, *p* = 0.91). These findings suggest comparable detection rates between MRI performed within 24 h and those performed after 24 h of seizure onset, although substantial inter‐study variability limits definitive conclusions. The resulting forest plot is presented in Supplementary Material 6.

## 4 DISCUSSION

NCSE remains an electroclinical diagnosis; however, neuroimaging may play a key complementary role in cases with ambiguous EEG findings or clinical presentations. In clinical settings, MRI and CTP are the most commonly employed imaging modalities for supporting the diagnosis of NCSE. This meta‐analysis compared the detection rate of peri‐ictal abnormalities in patients with NCSE using MRI or CTP. MRI showed a pooled abnormality rate of 50.0%, whereas CTP demonstrated a significantly higher rate of 79.3%. However, it is important to note that peri‐ictal abnormalities detected by MRI and CTP reflect distinct pathophysiological mechanisms. Although CTP primarily captures cerebral hyperperfusion secondary to increased metabolic demand,^17^, ^42^, ^43^ MRI abnormalities—such as diffusion restriction or hyperintensities in fluid‐attenuated inversion recovery (FLAIR)—reveal peri‐ictal cytotoxic or vasogenic edema due to unmet metabolic demand, leading to cellular swelling that is often transient but may progress to neuronal injury.^44^ The ability of CTP to detect peri‐ictal hyperperfusion in the early stages of NCSE makes it a valuable tool for rapid diagnosis, particularly in emergency settings.^17^ On the other hand, PMAs, particularly when identified in DWI and FLAIR, have been linked to poor functional outcomes and increased mortality.^15^, ^18^, ^45^

Across the included CTP studies, the most consistently evaluated perfusion maps were cerebral blood flow (CBF), cerebral blood volume (CBV), and mean transit time (MTT). In the context of seizure activity, CBF and CBV were generally reported to increase, reflecting cortical hyperperfusion, whereas MTT was typically decreased, indicating faster transit of contrast due to elevated CBF.^17^, ^31^, ^32^, ^46^ Among these studies, Merli et al.^31^ reported the highest detection rate (95%) for peri‐ictal abnormalities using the reverse time‐to‐maximum (rTmax) map, achieving a sensitivity of 95% and an area under the receiver‐operating characteristic (AUROC) curve of 0.79 (95% CI: 0.69–0.89). These findings suggest that the selection and interpretation of specific CTP maps may influence the detection of peri‐ictal changes in NCSE.^47^

Further results of this meta‐analysis showed that among MRI modalities, DWI had a pooled peri‐ictal abnormality detection rate of ~51%, closely aligning with the overall MRI rate of 50.0%. FLAIR demonstrated a lower rate of 35.7%, whereas ASL showed the highest detection rate at 88.8%. These modality‐specific findings suggest that DWI abnormalities are commonly reported in NCSE, likely reflecting the widespread clinical availability and routine use of this sequence. In comparison, ASL may offer higher detection rates for peri‐ictal changes, although its current clinical use remains limited. Therefore, the selection of imaging sequences plays a crucial role in the detection of PMAs in NCSE. Although MRI undoubtedly offers a significant advantage in identifying clinically relevant lesions and underlying etiologies,^48^ its diagnostic value for detecting PMAs in NCSE is highly dependent on the specific sequences and imaging protocols employed.

Moreover, both ASL and CTP demonstrated high detection rates of peri‐ictal abnormalities in NCSE, with ASL achieving proportions up to 100%^26^, ^28^ in the included studies. The comparison between CTP and ASL showed no significant differences in detecting hyperperfusion, suggesting that both modalities can contribute similarly to the diagnosis of NCSE. ASL offers a promising alternative for assessing perfusion changes without the use of contrast agents or radiation exposure.^49^ Similarly, contrast‐enhanced MRI perfusion may represent a promising MRI modality; however, due to the limited number of studies reporting this sequence, a subgroup meta‐analysis was not feasible.^36^, ^39^ Nevertheless, when perfusion MRI modalities are not available, CTP is a good alternative in the investigation of NCSE. Overall, perfusion imaging demonstrates the highest detection rates; therefore, its integration in cases with equivocal EEG patterns—such as IIC—may enhance the detection of peri‐ictal changes and support the diagnosis of NCSE.

It is notable that the temporal dynamics of peri‐ictal imaging abnormalities may play a critical role in their detectability. Indeed, among the studies included in this meta‐analysis, time‐to‐CTP and time‐to‐MRI were not consistently reported and, if available, varied considerably, with MRI typically performed later than CTP. More specifically, CTP in most of the included studies was performed within 24 h, whereas time to MRI showed greater variability. Among the MRI studies reporting time to MRI, a subgroup meta‐analysis showed no significant differences in detection rates between MRI studies performed within 24 h and after 24 h. Although the precise timing of their appearance remains unclear, perfusion‐based changes—such as those captured by ASL or CTP—are thought to occur early, often within minutes of SE onset, and may resolve quickly thereafter.^45^, ^49^, ^50^ In contrast, structural changes visible on DWI or FLAIR are believed to appear later and might take longer to resolve, persisting for days to weeks.^14^, ^49^ Therefore, the time interval between SE onset and imaging—time‐to‐MRI or time‐to‐CTP—may influence detection rates across modalities.

## 5 LIMITATIONS

The main limitation of this meta‐analysis is the substantial heterogeneity observed across included studies, particularly within the MRI subgroup. This variability could be attributed to differences in study designs, imaging protocols, time to imaging (which was only available in less than half of the included studies), patient populations, and diagnostic criteria for NCSE. Moreover, data on the timing between imaging and EEG were inconsistently reported across studies. This lack of standardized reporting limits the ability to assess potential temporal influences on diagnostic accuracy and should be addressed in future research. Although subgroup meta‐analyses and sensitivity analyses were conducted to mitigate this variability, the lack of standardized imaging protocols across studies, particularly in the selection and use of MRI sequences such as DWI, FLAIR, and ASL, may have influenced the detection and reporting of peri‐ictal abnormalities. In addition, the included studies were both retrospective and prospective, each with typical challenges related to data collection, the methodology applied, and the reporting of findings. Finally, the real‐world feasibility of implementing advanced neuroimaging techniques during the acute phase of SE remains constrained by factors such as scanner availability, institutional protocols, and patient stability.

## 6 CONCLUSION

In conclusion, CTP demonstrates a higher detection rate of peri‐ictal abnormalities compared to conventional MRI protocols. However, when ASL sequences are employed, both CTP and ASL show comparable detection rates in identifying peri‐ictal changes. Integrating ASL into MRI protocols might enhance the detection of peri‐ictal abnormalities and aid in the diagnosis of NCSE. Furthermore, more prospective studies with standardized imaging protocols are warranted to better delineate imaging biomarkers associated with NCSE.

## AUTHOR CONTRIBUTIONS

Pilar Bosque‐Varela: Conceptualization, Methodology, Data Collection, Data Curation, Visualization, Writing – Original Draft, and Writing – Review & Editing. Giorgi Kuchukhidze: Writing – Review & Editing. Wiebke Hahn: Writing – Review & Editing. Clara Jünemann: Data Collection and Writing – Review & Editing. Lukas Machegger: Data Collection, Visualization, and Writing – Review & Editing. Leona Möller: Data Collection and Writing – Review & Editing. Johannes Pfaff: Data Collection and Writing – Review & Editing. Eugen Trinka: Data Collection and Writing – Review & Editing. Susanne Knake: Data Collection and Writing – Review & Editing. Panagiota‐Eleni Tsalouchidou: Conceptualization, Methodology, Data Collection, Data Curation, Visualization, Formal Analysis, Writing – Original Draft, and Writing – Review & Editing.

## FUNDING INFORMATION

This research received no specific funding.

## CONFLICT OF INTEREST STATEMENT

P.B.V. has received travel grants and honoraria from Union Chimique Belge (UCB), not related to the presented work. G.K. received research grants from Austrian Research Fund (FWF), and travel grants and honoraria from UCB, Jazz Pharmaceuticals, and Novartis, none related to the presented work. JARP reports: Grants: Vesalio. Honoraria/Speaker fees: Boehringer Ingelheim AG & Co. KG, Eli Lilly GmbH, Medtronic, Siemens Healthcare Diagnostics GmbH. Travel: Cerenovus (Johnson & Johnson Medical Products GmbH), Vesalio. E.T. has received personal honoraria for lectures and educational activities from EVER Pharma, Biopas, Marinus, Arvelle, Angelini, Alexion, Argenx, Medtronic, Biocodex, Bial‐Portela & Ca, NewBridge, GL Pharma, GlaxoSmithKline, Boehringer Ingelheim, LivaNova, Eisai, Epilog/Clouds of Care, UCB, Biogen, Sanofi, Jazz Pharmaceuticals, and Actavis; his institution received research grants from Biogen, UCB Pharma, Eisai, Red Bull, Merck, Bayer, The European Union, FWF Osterreichischer Fond zur Wissenschaftsforderung Bundesministerium für Wissenschaft und Forschung, and Jubiläumsfond der Österreichischen Nationalbank. S.K. received speakers' honoraria from Bial, Destin Arzneimittel, Eisai, Jazz Pharma, Merck Serono, and UCB, none of which are related to the presented work. P.T. has received research grants from the German Society for Epileptology (Otfrid‐Foerster Stipendium, Deutsche Gesellschaft für Epileptologie (DGfE)), as well as travel grants from UCB and Angelini, and honoraria for lectures from UCB, none of which are related to the presented work. All other authors report no conflicts of interest related to this work.

## ETHICS STATEMENT

Ethical approval was not required as this study is based exclusively on previously published data. We confirm that we have read the Epilepsia position on ethical publication and affirm that this report is consistent with those guidelines.

## Supporting information


Data S1.

## Data Availability

All data relevant to this study are included in the article and Supplementary Materials. Additional data may be made available upon request from the corresponding author.

## References

[1] Trinka E , Cock H , Hesdorffer D , Rossetti AO , Scheffer IE , Shinnar S , et al. A definition and classification of status epilepticus‐‐report of the ILAE task force on classification of status epilepticus. Epilepsia. 2015;56(10):1515–1523. 26336950 10.1111/epi.13121

[2] Lawson J , Triner W , Kluge B . Occipital lobe status epilepticus, a stroke mimic with novel imaging findings: a case report. Clin Pract Cases Emerg Med. 2022;6(3):212–215. 36049189 10.5811/cpcem.2022.1.55482 PMC9436493

[3] Kanazawa Y , Morioka T , Arakawa S , Furuta Y , Nakanishi A , Kitazono T . Nonconvulsive partial status epilepticus mimicking recurrent infarction revealed by diffusion‐weighted and arterial spin labeling perfusion magnetic resonance images. J Stroke Cerebrovasc Dis. 2015;24(4):731–738. 25724245 10.1016/j.jstrokecerebrovasdis.2014.09.026

[4] Machegger L , Bosque Varela P , Kuchukhidze G , Steinbacher J , Öllerer A , Prüwasser T , et al. Quantitative analysis of diffusion‐restricted lesions in a differential diagnosis of status epilepticus and acute ischemic stroke. Front Neurol. 2022;13:926381. 35873780 10.3389/fneur.2022.926381 PMC9301206

[5] Trinka E , Leitinger M . Management of Status Epilepticus, refractory status epilepticus, and super‐refractory status epilepticus. Continuum. 2022;28(2):559–602. 35393970 10.1212/CON.0000000000001103

[6] Shorvon S , Trinka E . Nonconvulsive status epilepticus and the postictal state. Epilepsy Behav. 2010;19(2):172–175. 20692208 10.1016/j.yebeh.2010.06.016

[7] Leitinger M , Trinka E , Zimmermann G , Beniczky S . Salzburg criteria for nonconvulsive status epilepticus: Details matter. Epilepsia. 2019;60(11):2334–2336. 31595496 10.1111/epi.16361 PMC6972514

[8] Leitinger M , Beniczky S , Rohracher A , Gardella E , Kalss G , Qerama E , et al. Salzburg consensus criteria for non‐convulsive status epilepticus – approach to clinical application. Epilepsy Behav. 2015;49:158–163. 26092326 10.1016/j.yebeh.2015.05.007

[9] Shorvon SD . Status epilepticus: its clinical features and treatment in children and adults. Curr Opin Neurol. 1994;7(2):113–120.7517278

[10] Leitinger M , Trinka E , Gardella E , Rohracher A , Kalss G , Qerama E , et al. Diagnostic accuracy of the Salzburg EEG criteria for non‐convulsive status epilepticus: a retrospective study. Lancet Neurol. 2016;15(10):1054–1062. 27571157 10.1016/S1474-4422(16)30137-5

[11] Hirsch LJ , Fong MWK , Leitinger M , LaRoche SM , Beniczky S , Abend NS , et al. American clinical neurophysiology Society's standardized critical care EEG terminology: 2021 version. J Clin Neurophysiol. 2021;38(1):1–29. 33475321 10.1097/WNP.0000000000000806 PMC8135051

[12] Rubinos C , Reynolds AS , Claassen J . The ictal–interictal continuum: to treat or not to treat (and how)? Neurocrit Care. 2018;29(1):3–8. Available from: https://www.embase.com/search/results?subaction=viewrecord&id=L619256170&from=export 29139014 10.1007/s12028-017-0477-5

[13] Bauer G , Gotwald T , Dobesberger J , Embacher N , Felber S , Bauer R , et al. Transient and permanent magnetic resonance imaging abnormalities after complex partial status epilepticus. Epilepsy Behav. 2006;8(3):666–671. 16503204 10.1016/j.yebeh.2006.01.002

[14] Bosque Varela P , Machegger L , Steinbacher J , Oellerer A , Pfaff J , McCoy M , et al. Brain damage caused by status epilepticus: a prospective MRI study. Epilepsy Behav. 2024;161:110081. 39489995 10.1016/j.yebeh.2024.110081

[15] Bosque Varela P , Machegger L , Oellerer A , Steinbacher JJ , McCoy M , Pfaff J , et al. Imaging of status epilepticus: making the invisible visible. A prospective study on 206 patients. Epilepsy Behav. 2023;141:109130. 36803874 10.1016/j.yebeh.2023.109130

[16] Bosque Varela P , Tabaee Damavandi P , Machegger L , Prüwasser T , Zimmermann G , Oellerer A , et al. Magnetic resonance imaging fingerprints of status epilepticus: a case‐control study. Epilepsia. 2024;65(6):1620–1630. 38507291 10.1111/epi.17949

[17] Giovannini G , Malagoli M , Turchi G , Miani A , Orlandi N , Vaudano AE , et al. Cortical and thalamic hyper‐perfusion in non‐convulsive status epilepticus. Relationship between perfusion CT patterns and Salzburg EEG criteria. Seizure. 2021;92:10–17. 34391029 10.1016/j.seizure.2021.08.002

[18] Bonduelle T , Ollivier M , Trin K , Thomas B , Daubigney A , Michel V , et al. Association of Peri‐ictal MRI abnormalities with mortality, Antiseizure medication refractoriness, and morbidity in status epilepticus. Neurology. 2023;100(9):e943–e953. 36443013 10.1212/WNL.0000000000201599 PMC9990431

[19] Giovannini G , Kuchukhidze G , McCoy MR , Meletti S , Trinka E . Neuroimaging alterations related to status epilepticus in an adult population: definition of MRI findings and clinical‐EEG correlation. Epilepsia. 2018;59(Suppl 2):120–127. 30129213 10.1111/epi.14493

[20] Page MJ , McKenzie JE , Bossuyt PM , Boutron I , Hoffmann TC , Mulrow CD , et al. The PRISMA 2020 statement: an updated guideline for reporting systematic reviews. Syst Rev. 2021;10(1):89. 33781348 10.1186/s13643-021-01626-4 PMC8008539

[21] PROSPERO: International prospective register of systematic reviews.

[22] Leitinger M , Gaspard N , Hirsch LJ , Beniczky S , Kaplan PW , Husari K , et al. Diagnosing nonconvulsive status epilepticus: defining electroencephalographic and clinical response to diagnostic intravenous antiseizure medication trials. Epilepsia. 2023;64(9):2351–2360. 37350392 10.1111/epi.17694

[23] Beniczky S , Hirsch LJ , Kaplan PW , Pressler R , Bauer G , Aurlien H , et al. Unified EEG terminology and criteria for nonconvulsive status epilepticus. Epilepsia. 2013;54(Suppl 6):28–29. 10.1111/epi.12270 24001066

[24] Wells GA , Shea B , O'Connell D , Peterson J , Welch V , Losos M . The Newcastle–Ottawa Scale (NOS) for assessing the quality of nonrandomised studies in meta‐analyses. 2013.

[25] R Core Team . R: a language and environment for statistical computing. Vienna, Austria: R Foundation for Statistical Computing; 2024. Available from: https://www.r‐project.org/

[26] Shimogawa T , Morioka T , Sayama T , Haga S , Kanazawa Y , Murao K , et al. The initial use of arterial spin labeling perfusion and diffusion‐weighted magnetic resonance images in the diagnosis of nonconvulsive partial status epileptics. Epilepsy Res. 2017;129:162–173. 28092848 10.1016/j.eplepsyres.2016.12.008

[27] Hormigo A , Liberato B , Lis E , DeAngelis LM . Nonconvulsive status epilepticus in patients with cancer: imaging abnormalities. Arch Neurol. 2004;61(3):362–365. 15023812 10.1001/archneur.61.3.362

[28] Ohtomo S , Otsubo H , Arai H , Shimoda Y , Homma Y , Tominaga T . Hyperperfusion in the thalamus on arterial spin labelling indicates non‐convulsive status epilepticus. Brain Commun. 2021;3(1):fcaa223. 33501426 10.1093/braincomms/fcaa223 PMC7811763

[29] Jaraba Armas S , Sala‐Padró J , Veciana M , Arroyo P , Pedro J , Mora J , et al. New‐onset non‐lesional aphasic status epilepticus. Clinical description, diagnostic clues, and treatment algorithm. Acta Neurol Scand. 2022;145(5):579–589. 35130366 10.1111/ane.13586

[30] Guven ME , Pazarci NK . Early MRI changes in status epilepticus: associations with seizure characteristics, EEG findings, and prognosis in patients without large lesions. Epileptic Disord. 2025;27:264–274. 39921592 10.1002/epd2.20338

[31] Merli E , Romoli M , Galluzzo S , Bevacqua L , Cece ES , Ricci G , et al. Pragmatic computerised perfusion diagnostics for non‐convulsive status epilepticus: a prospective observational study. J Neurol Neurosurg Psychiatry. 2024;95(5):471–476. 38041670 10.1136/jnnp-2023-332152

[32] Hauf M , Slotboom J , Nirkko A , von Bredow F , Ozdoba C , Wiest R . Cortical regional hyperperfusion in nonconvulsive status epilepticus measured by dynamic brain perfusion CT. AJNR Am J Neuroradiol. 2009;30(4):693–698. 19213823 10.3174/ajnr.A1456 PMC7051787

[33] Gonzalez‐Martinez A , Trillo S , Benavides‐Bernaldo de Quirós C , Casado‐Fernández L , De Toledo M , Barbosa‐Del Olmo A , et al. Predictors of perfusion computed tomography alterations in stroke mimics attended as stroke code. Eur J Neurol. 2021;28(6):1939–1948. Available from: https://www.embase.com/search/results?subaction=viewrecord&id=L2010779491&from=export 33609295 10.1111/ene.14783

[34] Katramados AM , Burdette D , Patel SC , Schultz LR , Gaddam S , Mitsias PD . Periictal diffusion abnormalities of the thalamus in partial status epilepticus. Epilepsia. 2009;50(2):265–275. 18717714 10.1111/j.1528-1167.2008.01736.x PMC2879152

[35] Cornwall CD , Dahl SM , Nguyen N , Roberg LE , Monsson O , Krøigård T , et al. Association of ictal imaging changes in status epilepticus and neurological deterioration. Epilepsia. 2022;63(11):2970–2980. 36054260 10.1111/epi.17404 PMC9826342

[36] Azman F , Tezer FI , Saygi S . Aphasic status epilepticus in a tertiary referral center in Turkey: clinical features, etiology, and outcome. Epilepsy Res. 2020;167:106479. 33038720 10.1016/j.eplepsyres.2020.106479

[37] Requena M , Sarria‐Estrada S , Santamarina E , Quintana M , Sueiras M , Rovira A , et al. Peri‐ictal magnetic resonance imaging in status epilepticus: temporal relationship and prognostic value in 60 patients. Seizure. 2019;71:289–294. 31499473 10.1016/j.seizure.2019.08.013

[38] Jabeen SA , Cherukuri P , Mridula R , Harshavardhana KR , Gaddamanugu P , Sarva S , et al. A prospective study of diffusion weighted magnetic resonance imaging abnormalities in patients with cluster of seizures and status epilepticus. Clin Neurol Neurosurg. 2017;155:70–74. 28267656 10.1016/j.clineuro.2017.02.013

[39] Verma RK , Abela E , Schindler K , Krestel H , Springer E , Huber A , et al. Focal and generalized patterns of cerebral cortical veins due to non‐convulsive status epilepticus or prolonged seizure episode after convulsive status epilepticus ‐ a MRI study using susceptibility weighted imaging. PLoS One. 2016;11(8):e0160495. 27486662 10.1371/journal.pone.0160495 PMC4972361

[40] Chen J , Jin L , Zhou X , Lu Q , Liu Q , Huang Y . Power spectrum analysis and outcomes of non‐convulsive status epilepticus: a single‐center study. Neurol Sci. 2023;44(1):287–295. 36175811 10.1007/s10072-022-06419-8

[41] Sarria‐Estrada S , Santamarina E , Quintana M , Pareto D , Sueiras M , Auger C , et al. Magnetic resonance imaging findings in focal‐onset status epilepticus. Eur J Neurol. 2022;29(1):3–11. 34390102 10.1111/ene.15065

[42] Schwartz TH . Neurovascular coupling and epilepsy: hemodynamic markers for localizing and predicting seizure onset. Epilepsy Currents. 2007;7(4):91–94. 17694162 10.1111/j.1535-7511.2007.00183.x PMC1941907

[43] Duncan R . Epilepsy, cerebral blood flow, and cerebral metabolic rate. Cerebrovasc Brain Metab Rev. 1992;4(2):105–121. 1627438

[44] Culleton S , Talenti G , Kaliakatsos M , Pujar S , D'Arco F . The spectrum of neuroimaging findings in febrile infection‐related epilepsy syndrome (FIRES): a literature review. Epilepsia. 2019;60(4):585–592. 30854647 10.1111/epi.14684

[45] Meletti S , Monti G , Mirandola L , Vaudano AE , Giovannini G . Neuroimaging of status epilepticus. Epilepsia. 2018;59(Suppl 2):113–119. 30160066 10.1111/epi.14499

[46] Gonzalez‐Martinez J , Mullin J , Vadera S , Bulacio J , Hughes G , Jones S , et al. Stereotactic placement of depth electrodes in medically intractable epilepsy: technical note. J Neurosurg. 2014;120(3):639–644. Available from: https://www.embase.com/search/results?subaction=viewrecord&id=L372518806&from=export 24405074 10.3171/2013.11.JNS13635

[47] Romoli M , Merli E , Galluzzo S , Muccioli L , Testoni S , Zaniboni A , et al. Hyperperfusion Tmax mapping for nonconvulsive status epilepticus in the acute setting: a pilot case‐control study. Epilepsia. 2022;63(10):2534–2542. 35793391 10.1111/epi.17359 PMC9796764

[48] Algethamy HM , Alzawahmah M , Young GB , Mirsattari SM . Added value of MRI over CT of the brain in intensive care unit patients. Can J Neurol Sci. 2015;42(5):324–332. 26059742 10.1017/cjn.2015.52

[49] Matsuura K , Maeda M , Okamoto K , Araki T , Miura Y , Hamada K , et al. Usefulness of arterial spin‐labeling images in periictal state diagnosis of epilepsy. J Neurol Sci. 2015;359(1–2):424–429. 26478131 10.1016/j.jns.2015.10.009

[50] Kutluay E , Beattie J , Passaro EA , Edwards JC , Minecan D , Milling C , et al. Diagnostic and localizing value of ictal SPECT in patients with nonconvulsive status epilepticus. Epilepsy Behav. 2005;6(2):212–217. 15710307 10.1016/j.yebeh.2004.12.001

