# Supplementary material for: Peri‐ictal imaging abnormalities in non‐convulsive status epilepticus: A systematic review and meta‐analysis comparing magnetic resonance imaging and computed tomography perfusion
Source: Epilepsia. 2025 Aug 19;66(12):4895–909. doi: 10.1111/epi.18604 (PMC12779322; doi:10.1111/epi.18604)
Supplement: Supplementary file 1 — Data S1. [file EPI-66-4895-s001.docx]

**Supplementary Material**

1. **Supplementary Material 1: Systematic Literature Search Strategy**

A systematic search was conducted in five electronic databases to identify studies reporting MRI and CT perfusion abnormalities in patients with non-convulsive status epilepticus (NCSE), ictal–interictal continuum meeting NCSE criteria (IIC/NCSE), or status epilepticus (SE) with separately reported NCSE data. The following databases were searched:

- PubMed (MEDLINE)
- Embase
- Scopus
- Web of Science
- Cochrane Library

Two separate searches were conducted:

1. Studies reporting Magnetic Resonance Imaging (MRI) findings in NCSE, IIC/NCSE, or SE with separately reported NCSE data
2. Studies reporting Computed Tomography (CT) Perfusion findings in NCSE, IIC/NCSE, or SE with separately reported NCSE data

No language or publication date restrictions were applied. The full search strategies for each database are detailed below.

- 1. **PubMed (MEDLINE) Search Strategy**

MRI in NCSE

("nonconvulsive status epilepticus" OR "non-convulsive status epilepticus" OR "NCSE" OR "electrographic status epilepticus" OR "ictal interictal continuum" OR "IIC" OR "status epilepticus")

AND

("Magnetic Resonance Imaging"[MeSH] OR "MRI" OR "Diffusion-Weighted Imaging" OR "DWI" OR "Fluid Attenuated Inversion Recovery" OR "FLAIR" OR "Arterial Spin Labeling" OR "ASL")

CT Perfusion in NCSE

("nonconvulsive status epilepticus" OR "non-convulsive status epilepticus" OR "NCSE" OR "electrographic status epilepticus" OR "ictal interictal continuum" OR "IIC" OR "status epilepticus")

AND

("Tomography, X-Ray Computed"[MeSH] OR "Computed Tomography Perfusion" OR "CT Perfusion" OR "CTP" OR "Cerebral perfusion" OR "Perfusion Imaging")

- 1. **Embase Search Strategy**

MRI in NCSE:

('nonconvulsive status epilepticus' OR 'non-convulsive status epilepticus' OR 'NCSE' OR 'electrographic status epilepticus' OR 'ictal interictal continuum' OR 'IIC' OR 'status epilepticus')

AND

('magnetic resonance imaging'/exp OR 'MRI' OR 'diffusion-weighted imaging' OR 'DWI' OR 'fluid attenuated inversion recovery' OR 'FLAIR' OR 'arterial spin labeling' OR 'ASL')

CT Perfusion in NCSE:

('nonconvulsive status epilepticus' OR 'non-convulsive status epilepticus' OR 'NCSE' OR 'electrographic status epilepticus' OR 'ictal interictal continuum' OR 'IIC' OR 'status epilepticus')

AND

('computed tomography'/exp OR 'Computed Tomography Perfusion' OR 'CT Perfusion' OR 'CTP' OR 'Cerebral perfusion' OR 'Perfusion Imaging')

- 1. **Scopus Search Strategy**

MRI in NCSE:

(TITLE-ABS-KEY("nonconvulsive status epilepticus" OR "non-convulsive status epilepticus" OR "NCSE" OR "electrographic status epilepticus" OR "ictal interictal continuum" OR "IIC" OR "status epilepticus"))

AND

(TITLE-ABS-KEY("Magnetic Resonance Imaging" OR "MRI" OR "Diffusion-Weighted Imaging" OR "DWI" OR "Fluid Attenuated Inversion Recovery" OR "FLAIR" OR "Arterial Spin Labeling" OR "ASL"))

CT Perfusion in NCSE:

(TITLE-ABS-KEY("nonconvulsive status epilepticus" OR "non-convulsive status epilepticus" OR "NCSE" OR "electrographic status epilepticus" OR "ictal interictal continuum" OR "IIC" OR "status epilepticus"))

AND

(TITLE-ABS-KEY("Computed Tomography Perfusion" OR "CT Perfusion" OR "CTP" OR "Cerebral perfusion" OR "Perfusion Imaging"))

- 1. **Web of Science Search Strategy**

MRI in NCSE:

TS=("nonconvulsive status epilepticus" OR "non-convulsive status epilepticus" OR "NCSE" OR "electrographic status epilepticus" OR "ictal interictal continuum" OR "IIC" OR "status epilepticus")

AND

TS=("Magnetic Resonance Imaging" OR "MRI" OR "Diffusion Weighted Imaging" OR "DWI" OR "Fluid Attenuated Inversion Recovery" OR "FLAIR" OR "Arterial Spin Labeling" OR "ASL")

CT Perfusion in NCSE:

TS=("nonconvulsive status epilepticus" OR "non-convulsive status epilepticus" OR "NCSE" OR "electrographic status epilepticus" OR "ictal interictal continuum" OR "IIC" OR "status epilepticus")

AND

TS=("Computed Tomography Perfusion" OR "CT Perfusion" OR "CTP" OR "Cerebral perfusion" OR "Perfusion Imaging")

- 1. **Cochrane Library Search Strategy**

MRI in NCSE:

(nonconvulsive status epilepticus OR non-convulsive status epilepticus OR NCSE OR electrographic status epilepticus OR ictal interictal continuum OR IIC OR status epilepticus)

AND

("Magnetic Resonance Imaging" OR MRI OR "Diffusion-Weighted Imaging" OR DWI OR "Fluid Attenuated Inversion Recovery" OR FLAIR OR "Arterial Spin Labeling" OR ASL)

CT Perfusion:

(nonconvulsive status epilepticus OR non-convulsive status epilepticus OR NCSE OR electrographic status epilepticus OR ictal interictal continuum OR IIC OR status epilepticus)

AND

("Computed Tomography Perfusion" OR "CT Perfusion" OR "CTP" OR "Cerebral perfusion" OR "Perfusion Imaging")

1. **Supplementary Material 2:**

**Table S2.1: Additional clinical characteristics of the MRI Population in Included Studies**

| **Study** | **LPDEEG** | **Etiology** | **Mortality rate (%)** | **Good functional outcome** | **Reversibility -neuroimaging changes** |
| --- | --- | --- | --- | --- | --- |
| Jaraba Aramas et al | NA | Stroke: 20% | 48% | 16% | NA |
|  |  | Autoimmune/inflammatory: 40% |  |  |  |
|  |  | Toxic-metabolic: 24% |  |  |  |
|  |  | Unknown: 4% |  |  |  |
| Katramados et al | 100% | Pre-existing epilepsy: 14% | NA | NA | 0/3 (0%) |
|  |  | Ischemia: 14% |  |  |  |
|  |  | Hemorrhage: 6% |  |  |  |
|  |  | Malignancy: 28% |  |  |  |
|  |  | Infection: 3% |  |  |  |
|  |  | Hypoxia: 3% |  |  |  |
|  |  | Unknown 33% |  |  |  |
| Sarria-Estrada et al | NA | Cerebral neoplasm: 27% | NA | NA | NA |
|  |  | Toxic/drug overdose: 15% |  |  |  |
|  |  | Chronic cerebrovascular disease or traumatic brain injury: 13% |  |  |  |
|  |  | Unknown cause:12% |  |  |  |
|  |  | Non-adherence:10% |  |  |  |
|  |  | Acute stroke:10% |  |  |  |
|  |  | Acute infection: 5%, |  |  |  |
|  |  | Inflammatory/autoimmune: 5% and metabolic: 3% |  |  |  |
| Chen et al | NA | Encephalitis: 40% | NA | mRs at discharge: 2.91±1.68 | 25% in 3 days |
|  |  | Metabolic encephalopathy: 10% |  |  |  |
|  |  | Stroke: 2% |  |  |  |
|  |  | Creutzfeldt-Jakob Disease: 6% |  |  |  |
|  |  | Dementia: 4% |  |  |  |
|  |  | Leukoencephalopathy: 2% |  |  |  |
|  |  | Epilepsy: 35% |  |  |  |
|  |  | Moderate to heavy drinkers for more than 40 years : 4% |  |  |  |
|  |  | Heavy drinkers for more than 30 years 4: 8% |  |  |  |
|  |  | Perinatal hypoxic ischemic encephalopathy: 2% |  |  |  |
|  |  | Cryptogenic epilepsy: 19% |  |  |  |
| Ohtomo et al | 71% | Acute cerebral hemorrhage: 100% | NA | NA | NA |
| Giovannini et al | 28% | Tumor CNS: 21% | NA | NA | 44% |
|  |  | Stroke: 20%, |  |  |  |
|  |  | Autoimmune/inflammatory: 4%, |  |  |  |
|  |  | Post-traumatic: 4% |  |  |  |
|  |  | Toxic-metabolic: 9% |  |  |  |
|  |  | Unknown: 6% |  |  |  |
| Shimogawa et al. | 20% | Stroke: 13%, | NA | 67% | 73% |
|  |  | Epilepsy related lesions: 7%, |  |  |  |
|  |  | Unknown: 33% |  |  |  |
|  |  | Post-traumatic: 13% |  |  |  |
| Requena et al | 28% | NA | NA | 55% | NA |
| Guven et al | NA | NA | NA | NA | NA |
| Jabeen et al | 33% | Stroke: 13% | NA | NA | 80% |
|  |  | Toxic-metabolic: 20% |  |  |  |
|  |  | Unknown: 53% |  |  |  |
|  |  | Post-traumatic: 13%, |  |  |  |
| Azman et al | 56% | Tumor CNS: 38% | NA | NA | NA |
|  |  | Stroke: 37%, |  |  |  |
|  |  | Autoimmune/inflammatory: 6% |  |  |  |
|  |  | Toxic-metabolic: 12%, |  |  |  |
|  |  | Unknown: 6% |  |  |  |
| Verma et al | NA | Tumor CNS: 29% | NA | NA | NA |
|  |  | Stroke: 7% |  |  |  |
|  |  | Unknown: 50% |  |  |  |
|  |  | Post-traumatic: 7% |  |  |  |
| Bonduelle et al | NA | NA | NA | NA | NA |
| Hormigo et al | NA | Tumor CNS: 100% | 25% | NA | 75% |
| Cornwall et al | NA | NA | NA | NA | NA |

Abbreviations: LPD: lateralized periodic discharges, CNS: central nervous system, NA: not applicable, mRs: modified Rankin score

**Table S2.2: Additional clinical characteristics of the CTP Population in Included Studies**

| **Study** | **LPDs-EEG** | **Etiology** | **Mortality rate (%)** | **Good functional outcome** | **Reversibility -neuroimaging changes** |
| --- | --- | --- | --- | --- | --- |
| Merli et al | NA | Progressive: 5% | NA | NA | NA |
|  |  | Remote: 71% |  |  |  |
|  |  | Symptomatic: 19% |  |  |  |
|  |  | Unknown: 5% |  |  |  |
| Gonzalez-Martinez et al | NA | NA | NA | NA | NA |
| Giovaninni et al | NA | Tumor CNS: 19% | NA | NA | NA |
|  |  | Stroke: 47% |  |  |  |
|  |  | Autoimmune/inflammatory:5% |  |  |  |
|  |  | Toxic-metabolic: 9% |  |  |  |
|  |  | Unknown: 5% |  |  |  |
| Hauf et al | NA | Stroke: 56% | 44% | NA | NA |
|  |  | Autoimmune/inflammatory: 11% |  |  |  |
|  |  | Post-traumatic: 22% |  |  |  |
|  |  | Unknown: 11% |  |  |  |

Abbreviations: LPD: lateralized periodic discharges; NA: not applicable

# **Supplementary Material 3: NOS Summary Table – All Included Studies**

| Study | Selection | Comparability | Outcome |
| --- | --- | --- | --- |
| Jaraba Armas et al. 2021 | ★★★ | ★ | ★★★ |
| Katramados et al. 2010 | ★★★ | ★ | ★ |
| Sarria-Estrada et al. 2023 | ★★★ | ★★ | ★★★ |
| Chen et al. 2023 | ★★★ | ★ | ★★★ |
| Ohtomo et al. 2020 | ★★★ | ★ | ★ |
| Giovannini et al. 2018 | ★★★ | ★★ | ★ |
| Shimogawa et al. 2017 | ★★★ | ★★ | ★★★ |
| Requana et al. 2019 | ★★ | ★ | ★★★ |
| Guven & Pazarci 2025 | ★★★ | ★★ | ★★★ |
| Jabeen et al. 2017 | ★★★★ | ★ | ★★★ |
| Azman et al. 2020 | ★★★ | ★ | ★★★ |
| Verma et al. 2016 | ★★★★ | ★ | ★ |
| Bonduelle et al. 2023 | ★★★★ | ★★ | ★★★ |
| Hormigo et al. 2004 | ★ | ★ | ★★ |
| Cornwall et al. 2022 | ★★★★ | ★ | ★★★ |
| Merli et al. 2024 | ★★★★ | ★ | ★★★ |
| Gonzalez-Martinez et al. 2021 | ★★★★ | ★ | ★★★ |
| Giovannini et al. 2021 | ★★★★ | ★★ | ★★★ |
| Hauf et al. 2009 | ★★★★ | ★ | ★★★ |

1. **Supplementary Material 4: Funnel Plot for Assessment of Publication Bias in Included MRI Studies**


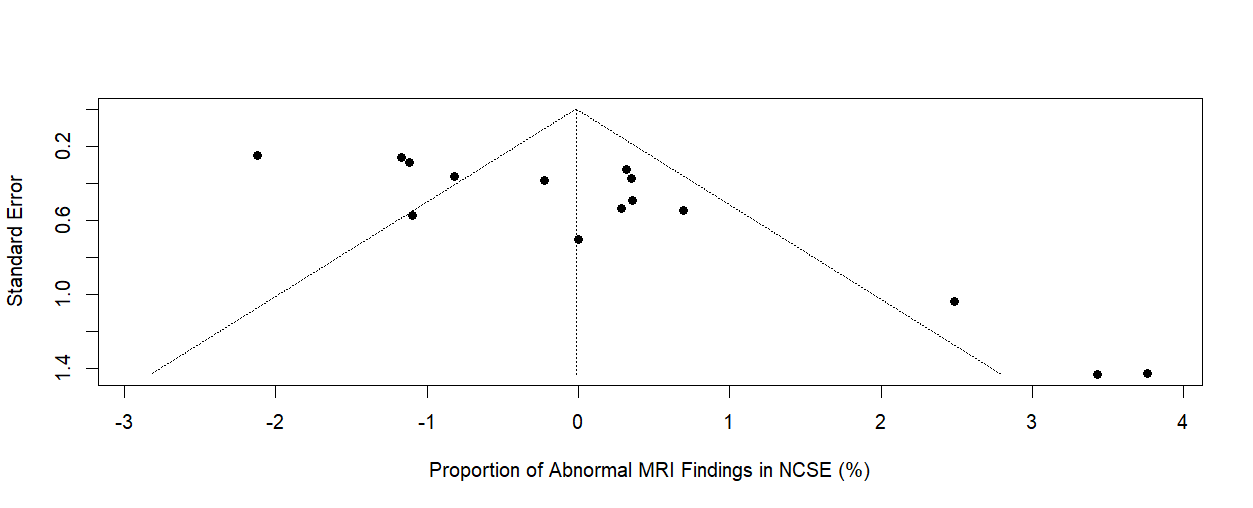


Supplementary Figure 4. Funnel plot illustrating the distribution of effect sizes in MRI studies assessing peri-ictal abnormalities in NCSE. Asymmetry suggests potential small-study effects or heterogeneity, though Egger’s test was not statistically significant (p = 0.2926)

1. **Supplementary Material 5: Leave-One-Out Sensitivity Analysis**

This table presents the pooled proportion after removing each study one at a time. The results show that no single study significantly altered the overall pooled estimate.

| Study Removed | Pooled Proportion | 95% CI (Lower) | 95% CI (Upper) |
| --- | --- | --- | --- |
| Jaraba Armas | -0.023 | -0.726 | 0.681 |
| Katramados | 0.072 | -0.632 | 0.777 |
| Sarria-Estrada | -0.022 | -0.730 | 0.685 |
| Chen | 0.030 | -0.686 | 0.746 |
| Ohtomo | -0.178 | -0.767 | 0.412 |
| Giovannini | 0.091 | -0.471 | 0.652 |
| T. Shimogawa. | -0.161 | -0.762 | 0.440 |
| Requena | -0.019 | -0.729 | 0.691 |
| Guven | -0.172 | -0.773 | 0.429 |
| Jabeen | -0.056 | -0.745 | 0.633 |
| Azman | 0.077 | -0.614 | 0.767 |
| Verma | -0.016 | -0.720 | 0.688 |
| Bonduelle | 0.090 | -0.600 | 0.780 |
| Hormigo | 0.006 | -0.696 | 0.709 |
| Cornwall | 0.093 | -0.593 | 0.779 |

1. **Supplementary Material 6:**

**
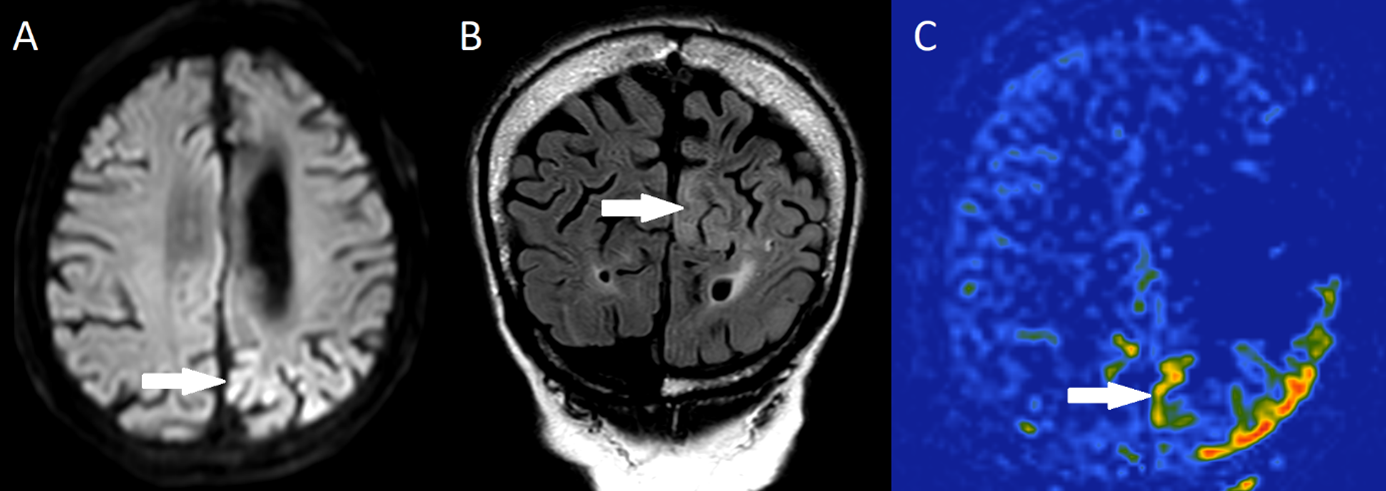
**

Example case: 64-year-old man with a focal (aphasic) NCSE. Etiology: encephalitis five years prior to this admission (Known remote etiology). Clinical duration of a SE - three hours, and 2 hours later, an MRI was performed (Image-A: Diffusion-restricted lesion in the left parietal region. Image-B: Hyperintense signal in left parietal region. Image-C: Hyperperfusion in the left parieto-temporal region in ASL)

1. **Supplementary Material 7: Forest Plot Presenting Detection Rates of Peri-Ictal Abnormalities on DWI in Early (<24h) vs. Late (>24h) MRI Subgroups**


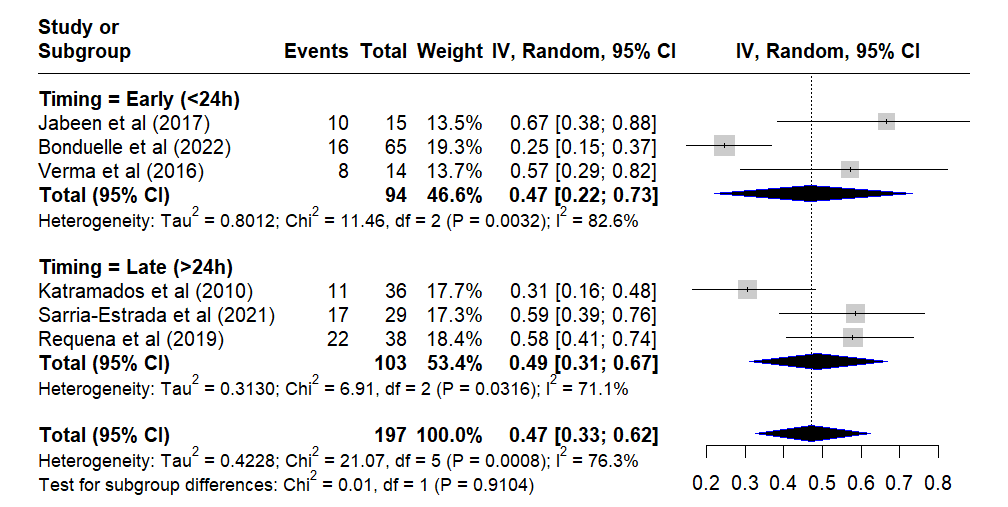


**Supplementary Figure S7:** Forest plot of subgroup meta-analysis comparing detection rates of peri-ictal MRI abnormalities on DWI between early (<24h) and late (>24h) MRI acquisition in NCSE patients. Pooled proportions and 95% confidence intervals are shown for each subgroup using a random-effects model. No significant difference was found between early and late groups (Q = 0.01, p = 0.91)
